# AMPK Activation by *Cimicifuga racemosa* Extract Ze 450 Is Associated with Metabolic Effects and Cellular Resilience against Age-Related Pathologies in Different Tissue Cell Types

**DOI:** 10.3390/pharmaceutics16030393

**Published:** 2024-03-13

**Authors:** Madeline Günther, Peter Schnierle, Thorsten Rose, Jonathan Schlegel, Georg Boonen, Jürgen Drewe, Eduardo Muñoz, Bernd L. Fiebich, Carsten Culmsee

**Affiliations:** 1 Institute of Pharmacology and Clinical Pharmacy, Biochemisch-Pharmakologisches Centrum Marburg, Philipps-University Marburg, Karl-von-Frisch-Straße 2, 35032 Marburg, Germany; 2 Center for Mind, Brain and Behavior, 35032 Marburg, Germany; 3 VivaCell Biotechnology GmbH, Ferdinand-Porsche-Str. 5, 79211 Denzlingen, Germany; 4 Medical Research, Max Zeller Soehne AG, 8590 Romanshorn, Switzerland; georg.boonen@zellerag.ch (G.B.);; 5 VivaCell Biotechnology España SL, Parque Científico Tecnológico Rabanales 21, 14014 Córdoba, Spain

**Keywords:** Ze 450, *Cimicifuga racemosa*, herbal extract, AMPK activation, metabolic effects, resilience, C2C12 myoblasts, HEPG2 liver cells, 3T3L1 adipocytes, HT22 neuronal cells, HTRF technology

## Abstract

*Cimicifuga racemosa* extracts (CREs) have gained well-established use for the treatment of menopausal symptoms such as hot flushes and excessive sweating, and weight gain. While the clinical effects of CREs have been well documented, the mechanisms underlying these effects are largely unknown. More recently, the metabolic effects of the CRE Ze 450 were demonstrated in cultured cells in vitro and in mouse models of obesity in vivo. At the molecular level, metabolic regulation, enhanced insulin sensitivity, and increased glucose uptake were linked to the activation of AMP-activated protein kinase (AMPK). Therefore, we tested the effects of Ze 450 on AMPK phosphorylation and thus activation in cells from different tissues, i.e., murine C2C12 myoblast cells, human HEPG2 liver cells, mouse HT22 neuronal cells, and in murine 3T3L1 adipocytes. Using a FRET-based HTRF-assay, we found that Ze 450 induced AMPK phosphorylation and the activation of this key enzyme of metabolic regulation in cells from various different tissues including C2C12 (muscle), HEPG2 (liver), HT22 (hippocampal), and 3T3-L1 (adipocyte) cells. In C2C12 muscle cells, enhanced AMPK activation was accompanied by reduced mitochondrial respiration and enhanced glucose uptake. Further, Ze 450 enhanced the resilience of the cells against oxidative death induced by ferroptosis inducers erastin or RSL3. Our findings suggest a general effect of *Cimicifuga racemosa* on AMPK activation in different tissues and across species. This may have a significant impact on expanded therapeutic applications of Ze 450, since AMPK activation and the related metabolic effects have been previously associated with anti-aging effects and the prevention of the metabolic syndrome.

## 1. Introduction

Menopause is a manifestation of the general aging process in women, with specific metabolic changes that aggravate menopausal symptoms, which are accelerated by estrogen depletion and associated neurotransmitter dysregulation. *Cimicifuga racemosa* extracts (CREs) such as Ze 450, an ethanolic dry extract derived from the rhizoma of *Cimicifuga racemosa* (L.) Nutt., have gained well-established use (EMA/265439/2018) for the treatment of menopausal symptoms including hot flushes and excessive sweating, weight gain, and mood swings [1,2]. While the clinical effects of CRE have been well documented, the molecular mechanisms underlying these effects on menopausal symptoms are largely unknown.

More recently, the metabolic effects of Ze 450 were demonstrated in vitro and in vivo, including effects in cultured HT22 neuronal cells and HEPG2 liver cells, in mouse models of obesity, and in postmenopausal women [3,4,5,6]. These metabolic effects of Ze 450 resulted in reduced weight gain, enhanced glucose tolerance, and insulin sensitivity, pointing at an antidiabetic potential of the CRE in vivo [5,6]. Such metabolic effects by Ze 450 mitigate climacteric symptoms but may also modulate the aging process itself. We have recently demonstrated that Ze 450 significantly enhanced the cellular resilience of neuronal and liver cells against oxidative damage in model systems of ferroptosis in vitro, and increased life span and resistance to paraquat-induced oxidative stress in *C. elegans* whole organisms in vivo [3,4]. At the cellular level, the inhibition of mitochondrial respiration and a metabolic switch to glycolysis were identified as the major mechanism of Ze 450 effects on energy metabolism, cell proliferation, and cellular resilience to oxidative stress [3,4]. These results suggested that Ze 450 may contribute to cellular resistance against oxidative dysregulation and to the healthy aging of whole organisms through the inhibition of mitochondrial respiration.

At the molecular level, metabolic switches from mitochondrial respiration to glycolysis, enhanced insulin sensitivity, and increased glucose uptake were linked to the activation of AMP-activated protein kinase (AMPK) [1,6]. In response to the inhibition of mitochondrial ATP production and decreasing energy supply, the cellular AMP/ATP ratio increases, thereby leading to AMP-mediated AMPK activation. Activated AMPK inhibits anabolic pathways, enhances catabolic pathways, and increases the cellular uptake and turnover of glucose to compensate for decreasing energy supplies [7]. Therefore, AMPK activation in peripheral tissues has been associated with antidiabetic effects and either weight loss or weight stabilization. Pharmacological approaches involving such AMPK-dependent antidiabetic effects also including weight loss apply, for example, for the antidiabetic drug metformin. Further, increasing evidence exposed AMPK activation as a potential key mechanism underlying CRE effects on menopausal complaints [1,6]. Since the multiple menopausal symptoms affect different organ systems [8,9] and are affected by CREs in the nervous system, in muscle liver, and fat tissue [1], we hypothesized that similar molecular mechanisms involving AMPK activation and according metabolic effects should be effective in cells from different tissues. Therefore, we tested the effects of Ze 450 on AMPK phosphorylation in cells from different tissues, i.e., murine C2C12 myoblast cells, human HEPG2 liver cells, and in murine 3T3L1 adipocytes with regard to the regulation of energy metabolism and glucose tolerance. Further, we evaluated the regulation of AMPK phosphorylation by Ze 450 in murine neuronal HT22 cells to verify AMPK activation as a general molecular mechanism across different tissues and organs. In turn, we also evaluated whether the effects of Ze 450 on mitochondrial respiration, energy metabolism, and cellular resilience to oxidative stress, as previously established in neuronal and liver cells [3,4], were also detectable in C2C12 muscle cells.

## 2. Materials and Methods

### 2.1. Extract

The ethanolic (60% *v*/*v*) *Cimicifuga racemosa* dry extract Ze 450 was manufactured from dried roots and rhizomes and obtained from Max Zeller and Soehne AG (Romanshorn, Switzerland), and thus, the indicated doses applied in this study contain 75% native extract. Ze 450 was dissolved in DMSO or in 60% ethanol (*v*/*v*) (Carl Roth GmbH, Karlsruhe, Germany) as indicated for the individual experiments. Ze 450 conforms to the herbal preparation B, which was granted with a well-established use status by a European Union herbal monograph on *C. racemosa* of the Herbal Medicinal Product Committee (HMPC) in 2010. The high-performance liquid chromatography (HPLC) fingerprint of the Ze 450 batch used is depicted in Figure S1.

### 2.2. Chemicals

AICAR, metformin, A769662, and rosiglitazone were purchased from Cayman (distributed by Biomol, Hamburg, Germany) in the highest purity available.

### 2.3. Cell Lines

C2C12 is an immortalized mouse myoblast cell line that is widely used as an in vitro model of skeletal muscle cell biology [10,11]. 3T3-L1 is a subclonal cell line used to study adipose tissue-related functions [12,13]. The mouse C2C12 myoblast and 3T3L1 adipocyte cell lines were obtained from LGC Standard (Barcelona, Spain) and grown in DMEM high glucose containing 10% fetal bovine serum (Bio & SELL GmbH, Feucht/Nürnberg, Germany), and 40 Units/mL penicillin, 40 µg/mL streptomycin, and 0.1 µg/mL fungizone (all obtained from Gibco, Thermo Fisher Scientific, Bonn, Germany). Cells were incubated at 37 °C in a humidified atmosphere with 5% CO_2_ in 75 cm^2^ cell culture flasks (Falcon, Heidelberg, Germany). Confluent monolayers were passaged routinely using trypsinization. After trypsinization, the cells were harvested and re-seeded into 6-(Western blot), 24-(HTRF), or 96-(cell viability and glucose uptake) well-plates (all from Greiner BioOne, Kremsmünster, Austria). On the next day, the medium was changed and after 1 h, the cells were stimulated for the respective experimental set-ups.

HepG2 (ATCC HB-8065, Manassas, VA, USA) cells were grown in Eagle’s minimum essential medium (EMEM, Merck KGaA, Germany) supplemented with 10% heat-inactivated fetal calf serum, 100 U/mL penicillin, and 100 mg/mL streptomycin, as described previously [4].

HT22 cells (kindly provided by David Schubert, Cellular Neurobiology Laboratory, Salk Institute for Biological Studies, La Jolla, CA, USA) were grown in Dulbecco’s modified Eagle medium (DMEM, Capricorn Scientific GmbH, Ebsdorfergrund, Germany), supplemented with 10% heat-inactivated fetal calf serum (Merck KGaA, Darmstadt, Germany), 100 U/mL penicillin, 100 mg/mL streptomycin (Capricorn Scientific GmbH, Ebsdorfergrund, Germany), and 2 mM L-glutamine (Merck KGaA, Darmstadt, Germany). To induce cell death, erastin (Calbiochem, Merck KGaA, Darmstadt, Germany) or RSL3 were added to the medium for the indicated amount of time (8–16 h).

### 2.4. Determination of Cell Viability

AlamarBlue assay. Cells were plated in 24-well or 96-well plates and treated with the respective test items for 24 h. Then, the cells were washed once with 100 μL PBS, and 100 μL of medium-AlamarBlue-Mix (90% medium, 10% AlamarBlue, DAL1025, Thermo Fisher Scientific, Bonn, Germany) was added to each well. The plate was incubated at 37 °C for 2 h in a humidified 5% CO_2_ atmosphere, and the color reaction was determined using a 96-well plate reader (excitation 544 nm, emission 590 nm).

MTT-Assay. Metabolic activity as an indicator of cell viability was quantified using the 3-(4,5-dimethylthiazol-2-yl)-2,5-diphenyltetrazolium bromide (MTT) assay. Viable and metabolically active cells convert MTT (Merck KGaA, Darmstadt, Germany), which was added at a concentration of 2.5 mg/mL for 1 h at 37 °C to the culture medium, into purple formazan. Absorbance was measured at 570 nm vs. 630 nm with FluoStar (BMG Labtech, Ortenberg, Germany) after dissolving in DMSO (Carl Roth GmbH, Karlsruhe, Germany).

Real time impedance measurements. Cell proliferation was analyzed in real time by measuring electrical impedance using the cCELLigence system (ACEA, Agilent, Santa Clara, CA, USA). Prior to cell seeding in a 96-well E-Plate (Omni Life Science GmbH, Bremen, Germany), the instrument was calibrated following the manufacturer’s instructions. Subsequently, the cells were allowed to grow for 24 h before Ze 450 treatment was administered. Changes in impedance measurements over time reflected alterations in cell number or cellular detachment from the plate. Data analysis was conducted using RTCA software 1.2 (Roche Diagnostics, Penzberg, Germany), which normalized the proliferation curves of treated samples to 1 before generating real-time growth curves and cell index values.

LDH Assay. Lactate dehydrogenase (LDH) is a soluble stable cytosolic enzyme present in many cell types that is rapidly released into the cell culture medium upon disruption of the plasma membrane. Therefore, LDH is a widely used marker in cytotoxicity studies. Here, we used the LDH-Glo™ Cytotoxicity Assay (Promega, Mannheim, Germany) according to the manufacturer’s protocol. In brief, C2C12 cells were plated in 96-well plates (2 × 10^4^ cells per well) and treated with the respective test items for 24 h. Then, 10 μL supernatant was removed and 40 μL LDH Storage Buffer added. Then, LDH Detection Reagent (containing Lactate, NAD+, Reductase, Reductase Substrate and Ultra-Glo™ rLuciferase) was added at a ratio of 1:1 (50 µL), and after 30 min of incubation, luminescence was measured using Victor X (Perkin Elmer, Rodgau, Germany). The luminescent signal generated is proportional to the amount of LDH in the sample.

### 2.5. Determination of AMPK Activation

To analyze AMPK phosphorylation and thus activation, a pAMPK and total AMPK HTRF assay was used (Cisbio, distributed by Perkin Elmer/Revvity GmbH, Hamburg Germany). Using HTRF technology, endogenous levels of total AMPK and AMPK phosphorylated at Thr 172 can be detected in cells after the lysis of the cell membrane in a sandwich assay format using 2 different specific antibodies labelled either with Eu3+-cryptate (fluorescent donor antibody) or with d2 (fluorescent acceptor antibody). When the dyes are in close proximity, the excitation of the donor with a light source (flash lamp with an excitation filter having a maximum transmittance at 340 nm) triggers a Fluorescence Resonance Energy Transfer (FRET) towards the acceptor at 615 nm, which in turn fluoresces at a specific wavelength (665 nm). The specific signal modulates positively in proportion to total-AMPK or pAMPK (Thr172). To analyze pAMPK in various cell types as used here, the cells were seeded at 200,000 cells per mL and well in 24-well plates and treated with the indicated treatment scheme as described in the figures. The cells were then lysed by adding Cisbio supplemented lysis buffer and incubated for 30 min at RT while shaking. A total of 1 µL supplemented lysis buffer/1000 cells was used. After homogenization by pipetting up and down, 16 μL of lysate was transferred from the cell-culture plate to a small volume detection plate. As per the manufacturer’s protocol, a 4 μL premixed solution of the two antibodies was added and incubated overnight, before the ratio of 665/615 nm was determined using a PerkinElmer Victor X5 2030-0050 Multimode Plate Reader (PerkinElmer, Rodgau, Germany). The ratio of the acceptor and donor emission signals was calculated for each individual well: Ratio = ((Signal 665 nm)/(Signal 615 nm)) × 10^4^. The mean and standard deviation were worked out from the ratio replicates and the coefficient of variation (CV) was calculated according to the formula: CV (%) = (standard deviation/mean ratio) × 100. The negative control (1 × supplemented lysis buffer) was used to check the non-specific signal. The ratio of control lysate signal/non-specific signal should be greater than 2, which was fulfilled in all measurements.

### 2.6. pAMPK Western Blot

3T3-L1 preadipocytes were cultured until confluence. Then, the cells were switched to differentiation medium (DMEM containing 0,5 mM IBMX, 1 μM Dexamethasone and 10 μg/mL Insulin). After 2 days of incubation in differentiation medium, the cells were switched to DMEM containing 5 μg/mL insulin and they were incubated for another 2 days. Then, the medium was changed to DMEM containing 10% FCS and 1% antibiotics and replaced every 2 days. Differentiation to mature adipocytes was analyzed by monitoring the formation of lipid droplets. After 2 weeks of differentiation, the cells were treated either with increasing concentrations of Ze 450 or with metformin as positive control for 24 h. After the indicated treatment, the cells were washed with phosphate-buffered saline (PBS), and proteins were extracted in lysis buffer (50 mM Tris–HCl pH 7,5, 150 mM NaCl, 10% glycerol, 1% NP-40, 10 mM NaF, 1 mM Na3VO4, 10 μg/mL leupeptin, 1 μg/mL pepstatin and aprotinin, and 1 μL/mL saturated PMSF). Protein concentrations were determined using the bicinchoninic acid (BCA) protein assay kit (Thermo Fisher Scientific, Bonn, Germany). For Western blotting, thirty μg of proteins was boiled at 95 °C in Laemmli buffer and electrophoresed in 12% SDS/PAGE gels. The separated proteins were transferred to PVDF membranes (Merck Millipore, Darmstadt, Germany) using semi-dry blotting and blocked with non-fat milk in TBST buffer afterwards. The primary antibodies used were pAMPKα (Thr172) (40H9), Vinculin (E1E9V) (Cell Signaling Technology Inc., Danvers, MA, USA); total AMPKα (#ab80039) (Abcam, Cambridge, UK), and β-actin (Sigma-Aldrich, Madrid, Spain). Membranes were incubated with the appropriate horseradish peroxidase-conjugated secondary antibody and detected using a chemiluminescence system (GE Healthcare Europe GmbH, Freiburg, Germany). Protein extraction and Western blots were performed three times from independent experiments.

### 2.7. Statistics

For statistical evaluation, each parameter was compared with control values using multiple unpaired, two-sided *t*-tests without assuming homogeneity of variances. *p*-values were adjusted for multiplicity of testing using the Bonferroni–Holm correction [11]. This model was implemented through the “multipletest” module of the Statsmodels statistical Python package (version 0.14.0, Wilmington, DE, USA) [13].

**Figure 1:**
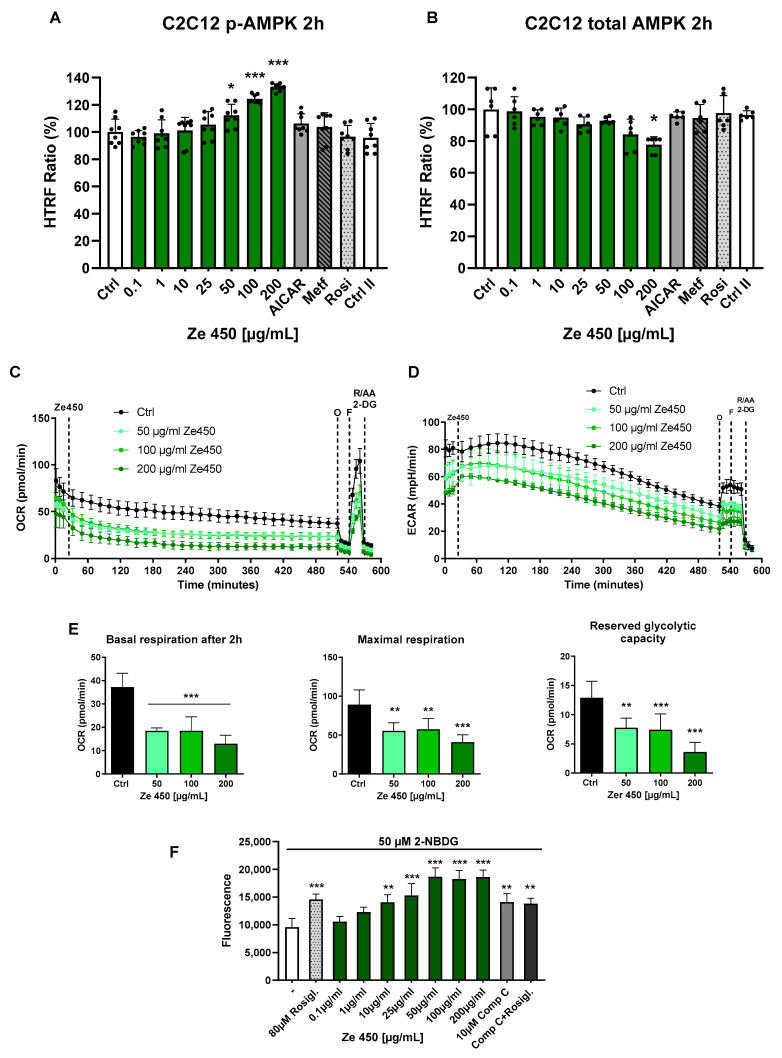
(**A**) Effects of *Cimicifuga racemosa* extract (CRE) ZE 450 on AMPK phosphorylation in C2C12 mouse muscle cells. Cells were stimulated with different doses of Ze 450 (0.1–200 µg/mL) and AICAR (2.5 mM), metformin (2.5 mM), and rosiglitazone (20 µM) as potential positive controls for 2 h. (**B**) Effects of CRE Ze 450 on protein levels of AMPK in C2C12 mouse muscle cells. Cells were stimulated with different doses of Ze 450 (0.1–200 µg/mL) and AICAR for 2 h. Total AMPK was determined using the HTRF assay. (**A**,**B**) The phosphorylation of AMPK was determined using the HTRF assay. * *p* < 0.05, *** *p* < 0.001, with respect to vehicle-treated cells (n = 3, multiple unpaired, two-sided *t*-tests with Bonferroni–Holm correction). (**C**) Metabolic effects of Ze 450 on mitochondrial respiration and glycolysis in C2C12 cells. Basal mitochondrial respiration (OCR) was detected using the Seahorse XF96 Flowmeter after exposure to Ze 450 (50–200 µg/mL). Further, the mitochondrial stress test was performed using oligomycin D (O, 3 µM), FCCP (F, 0.5 µM) and antimycin A (AA, 1 µM), rotenone (R 0.1 µM), and 2-deoxyglucose (2-DG, 50 mM) to test for ATP synthesis, maximal respiratory capacity, and basal mitochondrial respiration, respectively. (**D**) Extracellular acidification rate (ECAR) was measured to evaluate glycolytic activity under basal conditions and in the mitochondrial stress test. (**E**) Basal respiration was obtained from the difference in the OCR after 116–149.7 min and the non-mitochondrial respiration calculated as the response to AA/Rot/2-DG. Maximal respiration was determined by the OCR difference in the FCCP and the AA/Rot/2-DG response. Reserved glycolytic capacity was calculated by the difference in the FCCP- and oligomycin-mediated ECAR response. ** *p* < 0.01, *** *p* < 0.001 compared to the control cells (n = 4, ANOVA, Scheffe’s test). (**F**) Effects of Cimicifuga extract Ze 450 on glucose uptake in C2C12 mouse muscle cells. Cells were stimulated with different doses of Ze 450 (0.1–200 µg/mL) and rosiglitazone (80 µM) as positive control for 24 h. Glucose uptake was measured using 2-NBDG and subsequent fluorescence measurements. ** *p* < 0.01, *** *p* < 0.001, with respect to vehicle-treated cells (n = 4, multiple unpaired, two-sided *t*-tests with Bonferroni–Holm correction). Comp C.: Compound C.

Statistical data in Figures 1A–D, 3 and 4A–D are presented as mean ± standard deviation (S.D.) from three or more independent experiments. A statistical analysis of treatment groups was performed by analysis of variance (ANOVA) followed by Scheffé’s post hoc test. *p* < 0.05 was considered statistically significant. Calculations were conducted using Winstat standard statistical software 2012.0.96 (R. Fitch Software, Bad Krozingen, Germany).

## 3. Results

In order to test for the effects of Ze 450 on AMPK activation, we exposed C2C12 mouse myoblast cells to increasing concentrations of Ze 450 (0.1–200 µg/mL) and harvested cytosolic protein extracts after 2 h. As shown in Figure 1A, Ze 450 enhanced AMPK phosphorylation in a concentration-dependent manner at 50–200 µg/mL. The total protein levels of AMPK were not affected by Ze 450 or even significantly reduced at the highest concentrations of 200 µg/mL (Figure 1B). It is interesting to note that AICAR (2.5 mM) and metformin (2.5 mM) only slightly enhanced pAMPK levels at 2 h of incubation, suggesting that Ze 450-mediated AMPK activation occurs faster than effects mediated by the other AMPK stimulators (Figure 1A). Rosiglitazone (20 µM) did not affect AMPK phosphorylation and levels in C2C12 cells (Figure 1A,B).

We further evaluated the metabolic effects of Ze 450 on mitochondrial respiration and glycolysis in C2C12 cells. Ze 450 significantly reduced basal mitochondrial respiration (OCR) within 2 h of exposure (Figure 1C). Further, the mitochondrial stress test revealed that mitochondrial ATP production and respiratory reserve were significantly reduced in Ze 450-treated cells compared to controls (Figure 1E). In addition, ECAR representing glycolytic activity was also reduced in C2C12 cells treated with Ze 450 (Figure 1D). In the mitochondrial stress test, however, the oligomycin-induced increase in ECAR was lower (*p*-value) in Ze 450-treated cells compared to controls, suggesting that the glycolytic activity in these cells was almost maximal and could not be further enhanced (Figure 1E). AMPK activation and metabolic switches from mitochondrial respiration to glycolysis are associated with increased glucose uptake to compensate for the lower ATP synthesis rate per glucose molecule by glycolysis versus OXPHOS. As demonstrated in Figure 1F, Ze 450 enhanced cellular glucose uptake in C2C12 cells as measured by 2-NBDG in a concentration-dependent manner, and the Ze 450 effects even exceeded the effects of rosiglitazone (80 µM) that was added as a positive control.

Overall, Ze 450 increased AMPK phosphorylation (50–200 µg/mL) in the C2C12 mouse muscle cells in a concentration-dependent manner, and this was associated with a pronounced reduction in mitochondrial respiration and increased glucose uptake into the cells.

**Figure 2:**
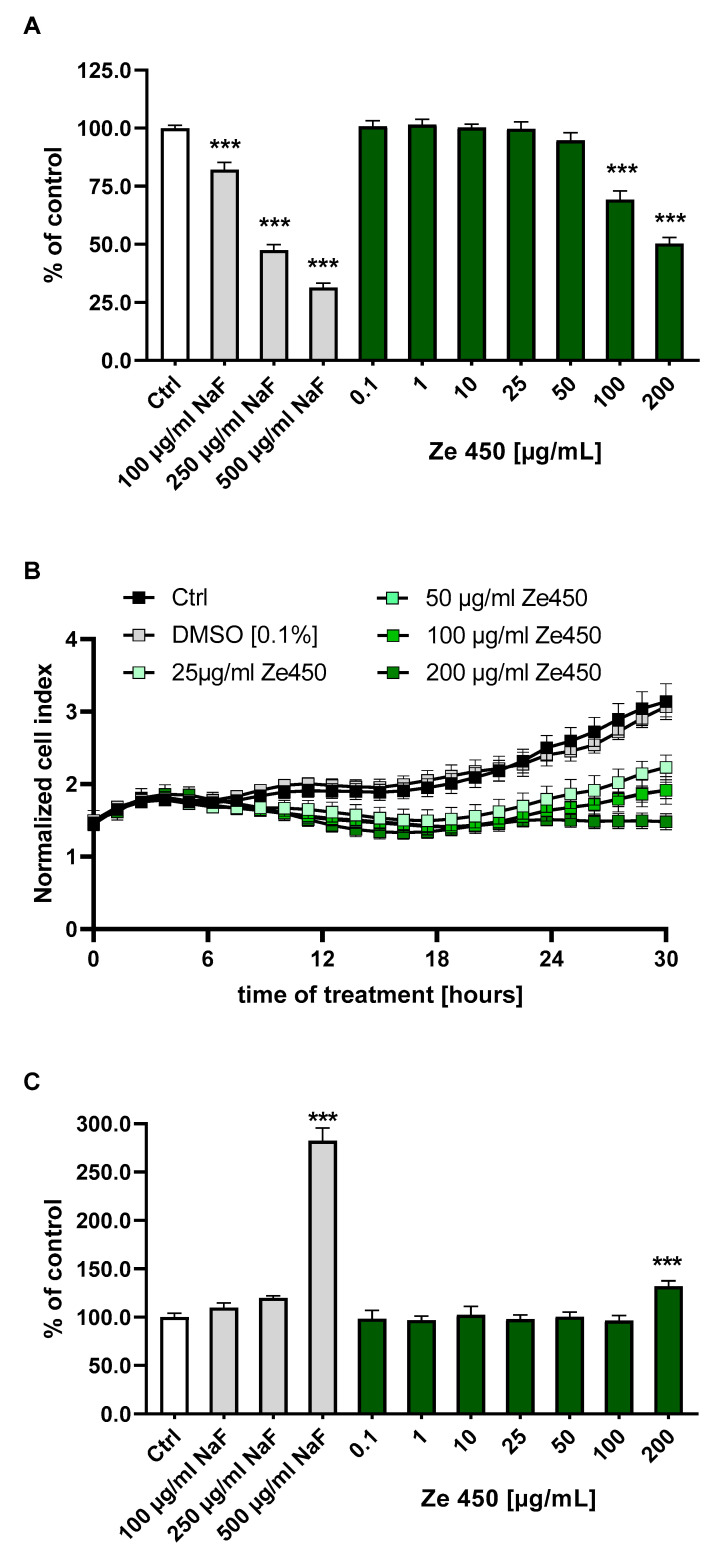
(**A**) Effects of *Cimicifuga racemosa* extract (CRE) Ze 450 on cell viability in C2C12 mouse muscle cells using the AlamarBlue/Formazan Assay. Cells were treated with different doses of Ze 450 (0.1–200 µg/mL) and sodium fluoride (NaF; 100–500 µg/mL) as toxic positive control for 24 h. Cell viability was measured by using AlamarBlue. *** *p* < 0.001 compared to vehicle-treated cells (n = 4, multiple unpaired, two-sided *t*-tests with Bonferroni–Holm correction). (**B**) C2C12 cells were exposed to Ze 450 (25–200 µg/mL) and long-term cell impedance measurements were performed using the xCELLigence system. (**C**) Effects of Ze 450 on cell viability in C2C12 mouse muscle cells using the LDH method. Cells were treated with different doses of Ze 450 (0.1–200 µg/mL) and sodium fluoride (NaF; 100–500 µg/mL) as toxic positive control for 24 h. Cell viability was measured by using AlamarBlue. *** *p* < 0.001, with respect to vehicle-treated cells (n = 4, multiple unpaired, two-sided *t*-tests with Bonferroni–Holm correction).

Next, we wanted to test whether the observed metabolic effects affected cell viability in C2C12 cells. The AlamarBlue assay revealed a significant reduction in metabolic activity at Ze 450 concentrations of 100 and 200 µg/mL, but these were not as pronounced as the reduced AlamarBlue staining after exposure to NaF (100–500 µg/mL) that was applied as a positive control (Figure 2A). Long-term cell impedance measurements using the xCELLigence system revealed that C2C12 cells exposed to Ze 450 showed reduced growth as detected through a lower increase in cell impedance over time at Ze 450 concentrations of 25–200 µg/mL (Figure 2B). However, the observed effects on cell metabolism and cell growth were not attributed to cell death as demonstrated by the LDH assay (Figure 2C). At high concentrations of NaF (500 µg/mL), LDH release was significantly increased, whereas none of the Ze 450 concentrations applied to the mouse muscle cells showed comparable toxic effects. At concentrations of 0.1–100 µg/mL of Ze 450, the detected LDH values were at control levels, and only at 200 µg/mL did the cells show a slight increase in LDH release (Figure 2C). Similar effects on metabolic effects using the AlamarBlue assay and on cell viability using the LDH assay were also observed with AICAR in the C2C12 cells (supplement Figures S2 and S3).

**Figure 3:**
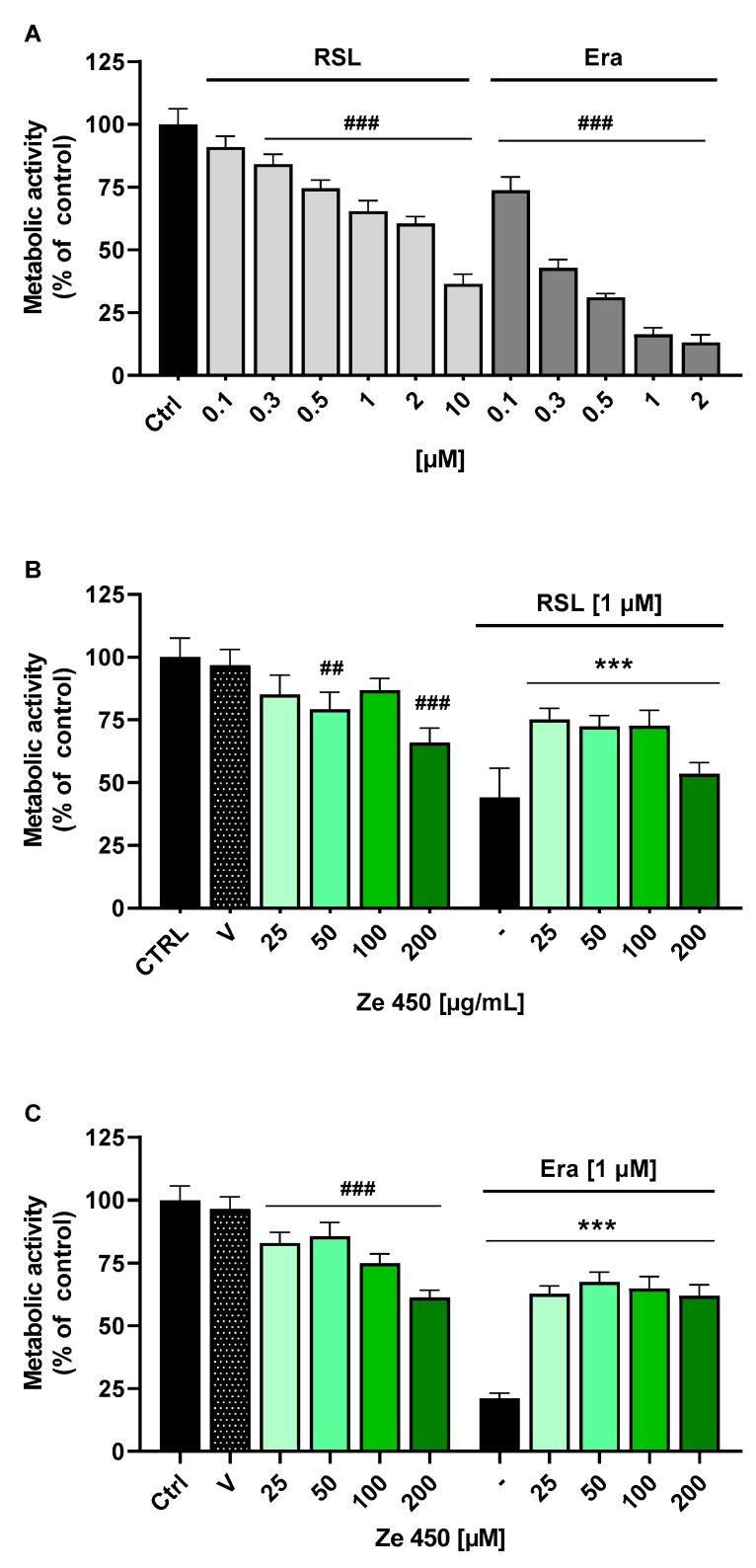
*Cimicifuga racemosa* extract (CRE) Ze 450 attenuates oxidative damage by ferroptosis. (**A**) C2C12 mouse muscle cells were exposed to ferroptosis inducers RSL (0.1–10 µM) and erastin (0.1–2 µM). C2C12 cells were sensitive to 0.3–10 µM RSL and 0.1–2 µM erastin as shown by the MTT assay, revealing reduced metabolic activities. Ze 450 protected the C2C12 cells from ferroptosis induced by 1 µM RSL (**B**) or 1 µM erastin (**C**) at concentrations of Ze 450 as low as 25 µg/mL. ## *p* < 0.01, ### *p* < 0.001 compared to untreated control; *** *p* < 0.001 compared to erastin or RSL control (n = 8, ANOVA, Scheffe’s test).

As shown so far, increasing concentrations of Ze 450 affected cell metabolism and cell proliferation, as detected in the measurements of OCR, using AlamarBlue staining and in the xCELLigence real-time impedance measurements, respectively. We next wanted to test whether these effects on AMPK activation and cell metabolism also resulted in altered sensitivity to cellular stress. Thus, we exposed C2C12 cells to ferroptosis, a form of non-apoptotic oxidative cell death that was induced here by two different ferroptosis inducers (FINs), i.e., erastin and RSL. As shown in Figure 3A, C2C12 mouse muscle cells were sensitive to both compounds. The MTT assay revealed reduced metabolic activities by 0.3–10 µM RSL and 0.1–2 µM erastin (Figure 3A). Ze 450 protected C2C12 cells from ferroptosis induced by 1 µM RSL (Figure 3B) or 1 µM erastin (Figure 3C) at concentrations of CRE as low as 25 µg/mL. These data showed that the metabolic effects detected before were associated with enhanced cellular resilience in the mouse muscle cells.

**Figure 4:**
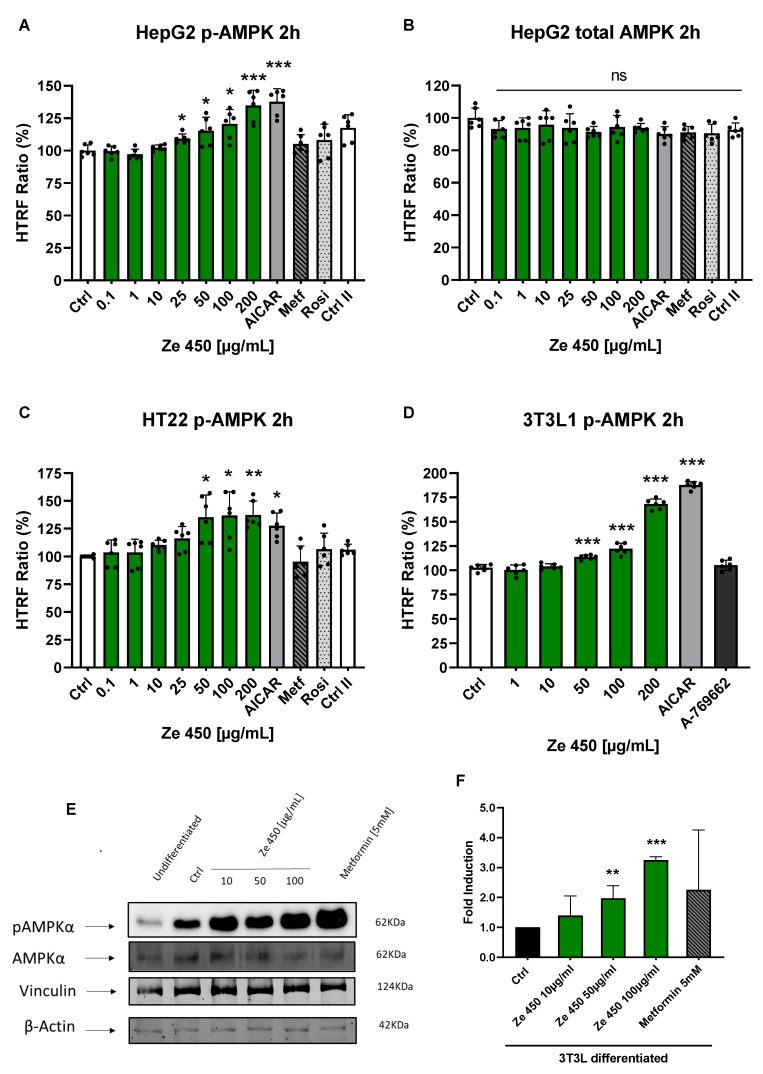
Effects of *Cimicifuga racemosa* extract (CRE) Ze 450 on pAMPK activation in HepG2 liver cells, HT22 neuronal cells, and 3T3 L1 adipocytes. (**A**) Cells were stimulated with different doses of Ze 450 (0.1–200 µg/mL) and AICAR (2.5 mM), metformin (2.5 mM), and rosiglitazone (20 µM) as potential positive controls for 2 h. The phosphorylation and thus activation of AMPK were determined using the HTRF assay. * *p* < 0.05, *** *p* < 0.001, with respect to vehicle-treated cells (n = 3, multiple unpaired, two-sided *t*-tests with Bonferroni–Holm correction). (**B**) Effects of Ze 450 on protein levels of AMPK in HepG2 liver cells. Cells were stimulated with different doses of Ze 450 (0.1–200 µg/mL) and AICAR (2.5 mM), metformin (2.5 mM), and rosiglitazone (20 µM) as potential positive controls for 2 h. Total AMPK was determined using the HTRF assay. ns (not significant) with respect to vehicle-treated cells (n = 3, multiple unpaired, two-sided *t*-tests with Bonferroni–Holm correction). (**C**) Effects of Ze 450 on pAMPK activation in HT22 mouse neuronal cells. Cells were stimulated with different doses of Ze 450 (0.1–200 µg/mL) and AICAR (2.5 mM), metformin (2.5 mM), and rosiglitazone (20 µM) as potential positive controls for 2 h. * *p* < 0.05, ** *p* < 0.01, with respect to vehicle-treated cells (n = 3, multiple unpaired, two-sided *t*-tests with Bonferroni–Holm correction). (**D**) Effects of Cimicifuga extract Ze 450 on the phosphorylation of AMPK in undifferentiated 3T3L1 pre-adipocytes. Cells were stimulated with different doses of Ze 450 (0.1–200 µg/mL) and AICAR (2.5 mM) and A769662 (100 µM) as positive control for 2 h. *** *p* < 0.001, compared to vehicle-treated cells (n = 3, ANOVA, Scheffe’s test). (**E**,**F**) Effects of ZE 450 on pAMPK in differentiated 3T3L1 adipocytes. Cells were stimulated with different doses of Ze 450 (0.1–200 µg/mL) and metformin (5 mM) as positive control for 2 h. pAMPK was determined using Western blot (**E**) and quantified (**F**) as described in the Methods section. Vinculin, β-actin, and total AMPK were used as loading controls. ** *p* < 0.01, *** *p* < 0.001 compared to vehicle-treated cells (n = 3; multiple unpaired, two-sided *t*-tests with Bonferroni–Holm correction).

Our previously published results suggested a similar effect of Ze 450 on cellular metabolism and resilience against oxidative stress in human HEPG2 liver cells and mouse HT22 neuronal cells. Therefore, we wanted to know whether these effects of Ze 450 were also associated with increased AMPK activation. We detected pAMPK and AMPK levels at 2 h after exposure of the different cell types to Ze 450 (Figure 4). Maximum effects on AMPK phosphorylation were achieved in HEPG2 liver cells at Ze 450 concentrations of 200 µg/mL, and these effects were as pronounced as the effects by AICAR (2.5 mM). Again, metformin (2.5 mM) and rosiglitazone (20 µM) failed to increase AMPK phosphorylation also in the liver cells within 2 h of exposure. Total AMPK levels were not affected by any of the compounds added to the cells (Figure 4B).

Similar increases in AMPK phosphorylation as in muscle and liver cells were also detected in neuronal HT22 cells (Figure 4C). In these neuronal cells, 50–200 µg/mL Ze 450 achieved enhanced AMPK phosphorylation, and this was comparable to 2.5 mM AICAR. Again, neither metformin nor rosiglitazone affected AMPK phosphorylation status after 2 h of incubation. Finally, we also tested the effects of Ze 450 in 3T3L adipocytes. In pre-adipocytes, 50–200 µg/mL Ze 450 increased AMPK phosphorylation as detected in the HTRF assay (Figure 4D). In differentiated 3T3L adipocytes, AMPK phosphorylation and thus activation was also increased as demonstrated by Western blot (Figure 4E,F). Metformin, applied at concentrations of 5 mM, also increased AMPK phosphorylation, but the results were not as consistent as with Ze 450 (Figure 4F).

## 4. Discussion

The present study demonstrates the potential of CRE Ze 450 to induce AMPK phosphorylation, i.e., the activation of this key enzyme of metabolic regulation in cells from various different tissues including C2C12 muscle cells, HEPG2 hepatoma cells, neuronal HT22 cells, and in 3T3-L1 adipocytes. In C2C12 muscle cells, enhanced AMPK activation was accompanied by reduced mitochondrial respiration and enhanced glucose uptake, suggesting metabolic alterations upon Ze 450 exposure. Notably, the metabolic alterations were also accompanied by reduced cell proliferation but did not cause apparent cytotoxic effects or cell death. In contrast, Ze 450 enhanced the resilience of the cells against oxidative death induced by ferroptosis inducers erastin or RSL3. A similar activation of AMPK by Ze 450 was also detected in other cell lines originating from liver, neural, or adipose tissue, suggesting a general effect of *Cimicifuga racemosa* on AMPK activation and the corresponding metabolic alterations in different tissues and across species.

The effects detected here point to a general mechanism of action of Ze 450 that affects cell metabolism with potential impact on major menopausal complaints such as weight gain and an increased risk of developing diabetes. Reduced estrogen levels are likely contributing to hyperphagia, hypometabolism, and adiposity in postmenopausal women, as similar effects have been also found in estrogen-receptor knockout mice [14,15]. In postmenopausal women, such metabolic changes may lead to obesity, insulin resistance, and dyslipidemia, which are also hallmark features of metabolic syndrome [16]. Hormone replacement therapy (HRT), but also Ze 450, efficiently and significantly improved postmenopausal symptoms, and CRE treatment exerted beneficial effects on metabolic parameters and weight similar to women receiving HRT [5]. In addition, studies in transgenic obese *ob/ob* mice [6] demonstrated positive effects of Ze 450 on weight gain and insulin sensitivity in male mice, demonstrating metabolic effects that occurred independently of the female hormone system. These are important findings since in human patients, HRT carries considerable risks and side effects attributed to the estrogen treatment. In fact, many women refuse HRT because of the expected side effects or the increased risk of developing hormone-dependent cancer, including breast cancer. Further, HRT can neither be applied to women with a history of cancer nor for the treatment of symptoms associated with chemotherapy-induced menopause in the adjuvant treatment of breast cancer. Further, the results obtained in experimental settings in male mice also imply the potential application of Ze 450 with positive effects on weight control in male individuals, i.e.**,** the therapy is not restricted to postmenopausal women. In fact, Ze 450 exerts therapeutic effects on menopausal complaints independent of estrogen receptor stimulation, since Ze 450 does not contain phytoestrogens or other components with known affinity to estrogen receptors. In addition, we have recently demonstrated that the metabolic effects of Ze 450 on mitochondrial respiration and cellular metabolism occurred independently of estrogen receptor activation [3].

One major concern when applying plant extracts, however, is potential cell toxicity with detrimental effects in the liver. Here, we confirmed that Ze 450 did not exert any cytotoxic effects to any of the cellular systems applied in the present study. At concentrations of Ze 450 that were required to affect mitochondrial respiration, cell metabolism, and AMPK activation, cellular proliferation was also significantly reduced. These metabolic effects, however, were not associated with cytotoxicity, and we have previously demonstrated in HEPG2 liver cells that these metabolic effects are fully reversible once Ze 450 is removed [4]. As shown in the models of ferroptosis, Ze 450 was rather cytoprotective in C2C12 muscle cells exposed to erastin or RSL3. This suggests that the observed metabolic effects, i.e.**,** the reduced mitochondrial respiration and associated effects on AMPK activation, glucose uptake, and glycolytic activation exerted protective effects very similar to previously reported effects in liver cells and in HT22 neuronal cells [3,4,17,18]. Such enhanced cellular resilience in the undifferentiated muscle cells exposed to Ze 450 may have an impact on muscle repair mechanisms, where undifferentiated muscle cells are needed for the regeneration of injured muscle tissue [19,20].

AMPK activation, as evaluated here in the different cell types through the quantification of AMPK phosphorylation at Thr172, occurred in a concentration-dependent manner at concentrations of Ze 450 that also mediated metabolic reprogramming and protective effects against oxidative stress in the C2C12 muscle cells, in the other cell types, and in *C. elegans*, as reported before [3,4]. The observed activation of AMPK also occurred transiently, and the kinetics were similar to AICAR-mediated effects but differed from effects exerted by the indirect AMPK activator metformin or the direct activator A796962. We and others previously reported protective effects by AICAR through AMPK activation in primary neuronal cells in models of glutamate toxicity and amyloid-beta-induced neurodegeneration [3,21,22]. The analysis of the kinetics of AMPK activation suggested that transient oscillating activation, as also observed here, is an important feature of protective effects mediated by AMPK, whereas the permanent long-term activation of AMPK rather mediated cytotoxicity [23]. According to these studies, protection through the transient activation of AMPK was mediated through glucose transporter 3 expression [24], whereas the pro-apoptotic activation of AMPK was attributed to the transcriptional activation of the proapoptotic Bcl-2 family member Bim [25]. Consequently, AMPK is regarded as a molecular switch that can determine protective versus cell death-inducing effects, depending on the kinetics and level of AMPK activation [23].

Whether Ze 450 directly activated AMPK, or did so indirectly through increasing AMP/ATP ratios in consequence to the reduced mitochondrial respiration and reduced ATP production, remains unclear. Our previous studies on protective effects by Ze 450 in models of ferroptosis demonstrated that reduced mitochondrial respiration enhanced the resilience of the organelles and the whole cells against oxidative damage triggered by accelerated lipid peroxidation. As shown previously, mitochondrial ROS production, mitochondrial fragmentation, and the loss of mitochondrial integrity significantly contribute to oxidative death by ferroptosis [26,27,28]. Here, we demonstrated similar protective effects of Ze 450 in C2C12 muscle cells and further showed AMPK activation in these cells associated with the observed metabolic alterations and resilience to oxidative damage. Further, the cells from other tissues, including liver cells and neuronal cells, also showed AMPK activation, and these cells were also protected from oxidative damage by Ze 450, as shown before [3,4].

Interestingly, pronounced effects of Ze 450 on AMPK activation were also observed in adipose tissue. This fits well with observations of positive effects of Ze 450 on body weight in transgenic obese mice [6,29] and in a retrospective study in postmenopausal women [5]. Further investigations revealed that 23-epi-26-deoxyactein, a major component of Ze 450, reduced adipogenesis in 3T3-L1 preadipocytes and prevented diet-induced obesity in C57BL/6 mice, similar to the component actein [29,30]. In the transgenic obese mouse model, Ze 450 also rescued glucose tolerance [6]. While such antidiabetic activity can be explained by metabolic effects exerted by Ze 450 in liver or muscle tissue, direct effects on fat tissue may also significantly contribute to effects on weight gain and, indirectly, on the development of diabetes. It is well established that the risk of developing diabetes increases with body weight gain, with fat tissue serving as a source for cytokine production, and low-grade chronic inflammation mediating insulin resistance in the liver and, hence, declining glucose tolerance. Moreover, previous studies reported decreased intra-abdominal fat tissue in ovariectomized rats treated with Cimicifuga extracts [31]. Furthermore, Ze 450 prevented the development of metabolic syndrome, cartilage degeneration, and osteoporosis in the ovariectomized rats, connecting the metabolic effects of CREs with further therapeutic effects on the consequences of estrogen deprivation resembling menopause [32,33,34,35]. Our data now connect the activation of AMPK in adipocytes with these previously reported effects of Ze 450 on adipose tissue, and the inhibition of metabolic syndrome and other menopausal pathologies.

Despite recent progress in the exploration of how AMPK mediates crosstalk between energy-sensing regions and tissues, i.e.**,** to increase food intake and body weight, thus restoring energy balance to the organism, the signals that can lead to AMPK activation are not universally conserved across different types of tissue [36,37]. Among others, it has been observed that leptin is capable of inhibiting AMPK in the hypothalamus, while it activates AMPK signaling in the muscle [38,39]. This represents an interesting dilemma for the development of therapeutics targeting AMPK activation. Strikingly, Ze 450 seems to act as a reliable activator of AMPK, which was demonstrated in different tissue cell types across our study, even though the underlying mechanism is still a matter of investigation.

*Cimicifuga racemosa* extract as an activator of AMPK at the cellular level is of significant interest and can be associated with an overall positive effect on aging related processes. In this context, several research approaches have revealed that the responsiveness of AMPK activation declines during the aging process. Ljubicic and Hood (2009) demonstrated that AMPK activation induced by muscle contractions was diminished in aged muscles, suggesting a decline in sensitivity to AMPK activation with aging [40,41]. Furthermore, treatments such as AICAR and physical exercise effectively increased AMPKα2 activity in the muscles of young rats but failed to elicit a similar response in old rats [42]. In a mouse model of cerebrovascular stroke, it was also observed that AMPK activation was significantly more pronounced in young mice than in old mice [43]. This decline in AMPK responsiveness has also been linked to various age-associated diseases, as evidenced by Qiang et al. [44], who reported impaired AMPK activation and suppressed glucose uptake in the skeletal muscles of aging rats. All these observations indicate that there is a clear deficiency in the sensitivity of AMPK activation in aged tissues, whereas studies with lower organisms have consistently demonstrated that increased AMPK activity can extend lifespan [45,46,47]. Finally, our data suggest that AMPK activation is a universal response of cells to Ze 450 from various tissues, as shown here for cells originating from liver, muscle, neuronal, and adipose tissue. These effects were independent of cell toxicity and, moreover, were accompanied by metabolic reprogramming. Notably, these metabolic effects were associated with mitochondrial and cellular resilience to oxidative stress. This may have a significant impact on the consideration of future therapeutic applications of Ze 450 since such metabolic effects and AMPK activation have been previously associated with anti-aging effects and the prevention of metabolic syndrome. Thus, Ze 450 may not only be considered for the treatment of postmenopausal complaints but also, beyond the application to elderly women, for the treatment or prevention of age-related diseases that involve metabolic impairments and the accumulation of oxidative stress. Since the therapeutic effects of Ze 450 do not involve estrogen receptor activation and target different tissues with beneficial effects on metabolism and cellular resilience, such applications may also be extended to both sexes.

## 5. Study Limitations

While our study provides valuable insights into the regulatory role of Ze 450 extract on AMP-activated protein kinase (AMPK) in different cell types, several limitations should be acknowledged. The use of immortalized cell lines, such as HepG2 (liver), C2C12 (muscle), 3T3-L1 (adipocytes), and HT22 (hippocampal), may not fully recapitulate the physiological environment found in vivo, since they lack the complexity of tissue microenvironments, intercellular interactions, and physiological regulation present in living organisms. Further limitations apply for potential sex differences which could not be mimicked in the studies performed in cultured cells. This has been shown to be an intriguing factor for AMPK activation and the sensing of whole-body energy status, since AMPK activation is integrated by peripheral signals, such as hormones, metabolites, and nutrition components, monitored in the hypothalamus and hindbrain, which in turn acts on peripheral tissues and/or organs by the release of glucagon, catecholamines, or corticosteroids, regulating hepatic glucose production, lipid metabolism, brown adipose tissue (BAT), thermogenesis, glucose homeostasis, and lipid oxidation in skeletal muscle. Additionally, herbal extract treatment in our experiments was limited to a defined time period (e.g., 24 h). Longer-term treatment studies would elucidate the sustained effects of AMPK modulation and potential adaptive responses over time. It also has to be taken into account that different cell types express AMPK to varying degrees and may respond differently to AMPK activation or inhibition; consequently, the effects of Ze 450 observed here can lead to various outcomes across different cellular and physiological contexts.

## Data Availability

Original research data are available on reasonable request.

## Supplementary Materials

The following supporting information can be downloaded at: https://www.mdpi.com/article/10.3390/pharmaceutics16030393/s1, Figure S1: HPLC fingerprint of the applied Ze 450 batch 1900026 with quantitative analysis for triterpene-glycosides; Figure S2: Effects of AICAR on cell viability in C2C12 mouse muscle cells using AlamarBlue/Formazan Assay; Figure S3: Effects of AICAR on cell viability in C2C12 mouse muscle cells using the LDH method.
