# Supplementary material for: AMPK Activation by Cimicifuga racemosa Extract Ze 450 Is Associated with Metabolic Effects and Cellular Resilience against Age-Related Pathologies in Different Tissue Cell Types
Source: Pharmaceutics. 2024 Mar 13;16(3):393. doi: 10.3390/pharmaceutics16030393 (PMC10975534; doi:10.3390/pharmaceutics16030393)
Supplement: Supplementary file 1 [file pharmaceutics-16-00393-s001.zip › pharmaceutics-2876325-supplementary.pdf]

Supplement Figures

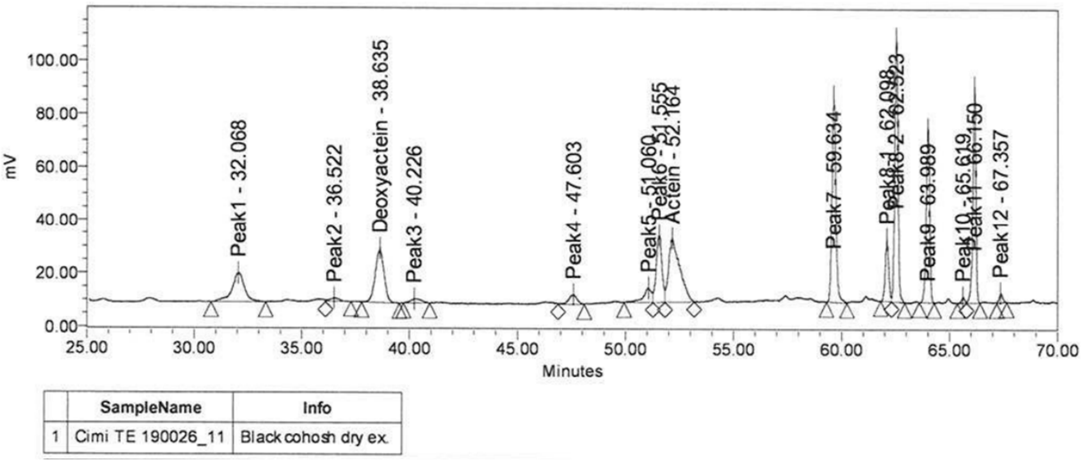


**Figure S1.** High-performance liquid chromatography (HPLC) fingerprint of the applied Ze 450 batch 1900026 with quantitative analysis for triterpene-glycosides.

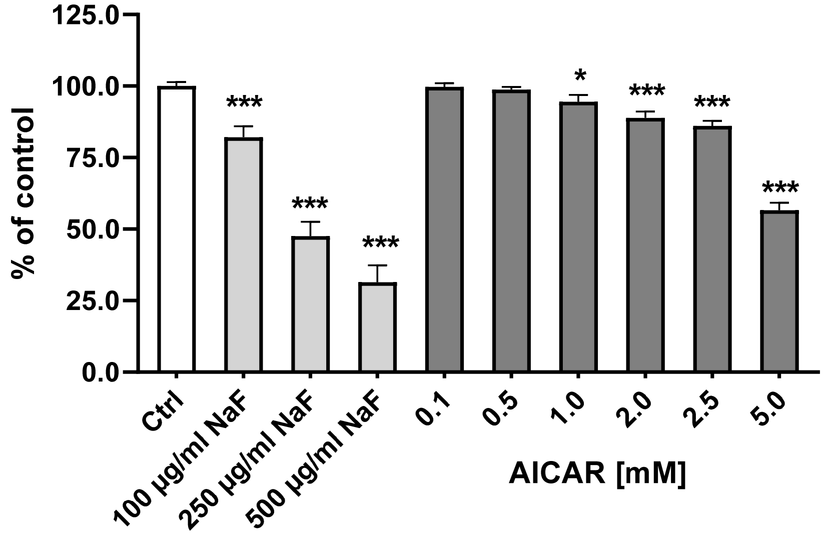


**Figure S2.** Effects of AICAR on cell viability in C2C12 mouse muscle cells using AlamarBlue/Formazan Assay. Cells were treated with different doses of AICAR (0.1–5 mM) and sodium fluoride (NaF; 100–500 µg/mL) as toxic positive control for 24 h. Cell viability was measured by using AlamarBlue. * $p < 0.05$, *** $p < 0.001$ compared to vehicle treated cells (n = 4, multiple unpaired, two-sided *t*-tests with Bonferroni-Holm correction).

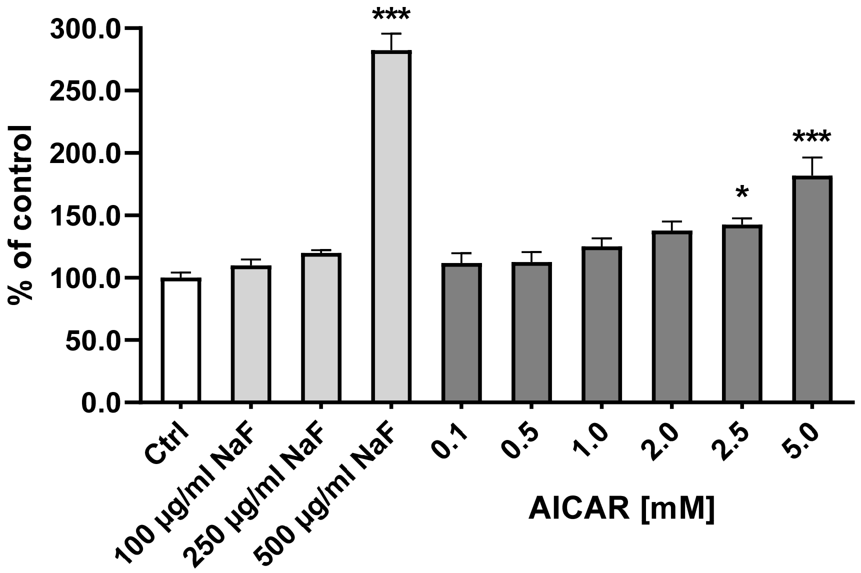


**Figure S3.** Effects of AICAR on cell viability in C2C12 mouse muscle cells using the LDH method. Cells were treated with different doses of AICAR (0.1–5 mM) and sodium fluoride (NaF; 100–500 µg/mL) as toxic positive control for 24 h. * $p < 0.05$, *** $p < 0.001$ compared to vehicle treated cells (n = 4; multiple unpaired, two-sided *t*-tests with Bonferroni-Holm correction).
